# Monogamy and high relatedness do not preferentially favor the evolution of cooperation

**DOI:** 10.1186/1471-2148-11-58

**Published:** 2011-03-04

**Authors:** Peter Nonacs

**Affiliations:** 1 Department of Ecology and Evolutionary Biology, University of California, Los Angeles, CA 90095, USA

## Abstract

**Background:**

Phylogenetic analyses strongly associate nonsocial ancestors of cooperatively-breeding or eusocial species with monogamy. Because monogamy creates high-relatedness family groups, kin selection has been concluded to drive the evolution of cooperative breeding (i.e., the monogamy hypothesis). Although kin selection is criticized as inappropriate for modeling and predicting the evolution of cooperation, there are no examples where specific inclusive fitness-based predictions are intrinsically wrong. The monogamy hypothesis may be the first case of such a flawed calculation.

**Results:**

A simulation model mutated helping alleles into non-cooperative populations where females mated either once or multiply. Although multiple mating produces sibling broods of lower relatedness, it also increases the likelihood that one offspring will adopt a helper role. Examining this tradeoff showed that under a wide range of conditions polygamy, rather than monogamy, allowed helping to spread more rapidly through populations. Further simulations with mating strategies as heritable traits confirmed that multiple-mating is selectively advantageous. Although cooperation evolves similarly regardless of whether dependent young are close or more distant kin, it does not evolve if they are unrelated.

**Conclusions:**

The solitary ancestral species to cooperative breeders may have been predominantly monogamous, but it cannot be concluded that monogamy is a predisposing state for the evolution of helping behavior. Monogamy may simply be coincidental to other more important life history characteristics such as nest defense or sequential provisioning of offspring. The differing predictive outcome from a gene-based model also supports arguments that inclusive fitness formulations poorly model some evolutionary questions. Nevertheless, cooperation only evolves when benefits are provided for kin: helping alleles did not increase in frequency in the absence of potential gains in indirect fitness. The key question, therefore, is not whether kin selection occurs, but how best to elucidate the differing evolutionary advantages of genetic relatedness versus genetic diversity.

## Background

Cooperatively breeding groups often have two defining characteristics: increased productivity over solitary individuals and differential within-group reproductive success or skew [1]. One puzzling feature of reproductive inequality is that subordinate roles appear to be willingly accepted. Kin selection is the dominant evolutionary paradigm for this apparent reproductive altruism [2-5]. In groups of relatives, an individual reducing its reproduction can be favored if this sufficiently benefits genetic kin. Increasingly, however, the relative importance of genetic relatedness for the evolution of reproductive skew is being questioned [1,6-8]. If groups are sufficiently successful, then individual genetic differences creating within-group reproductive skew can be evolutionarily stable [9]. Alternatively, parental manipulation can bias offspring to assume otherwise reproductively disadvantageous roles [10-12]. If such groups remain intact over generations of shared reproduction, then high relatedness may be a consequence and not a cause of cooperation. Indeed, Wilson and others argue kin selection acts more often as a 'dissolutive' than a binding force within groups [6-8].

The proposed alternatives to kin selection for reproductive skew have been challenged on both theoretical and empirical grounds [2-5]. The importance of kin selection in the evolution of cooperative breeding is argued to be particularly evident in its taxonomic distribution with respect to mating strategy. By Hamilton's rule, helping a relative is selectively favored when relatedness exceeds the cost/benefit ratio, or *r *>*c/b*. With monogamy, helping a parent rear full siblings is as valuable as rearing one's own offspring (*r *= 0.5 for both, where *r *is the probability of an allele being identical by descent through a common ancestor). Therefore, any reduction in cost or increase in benefit favors helping. In contrast, polygamy in terms of multiple fathers produces half sibs (*r *= 0.25) and cooperation would require halving costs or doubling benefits. Thus, kin selection predicts the evolution of cooperation ought to be most likely in species preadapted to maintain high relatedness between siblings (i.e., the monogamy hypothesis) [13,14]. Monogamy and high relatedness were first proposed to be critical in evolutionary transitions in social insects from cooperative breeding with totipotent individuals, to eusocial systems with morphological queen and worker castes [13,14]. Two recent phylogenetic analyses have extended the monogamy hypothesis to cooperative breeding, *per se*. Monogamy or lower levels of promiscuity are the most likely ancestral state for evolutionary transitions from solitary to social living in hymenoptera [15] and birds [16]. Conversely, lack of monogamy is hypothesized as evolutionarily constraining social degus (*Octodon degu*) from more elaborate sociality as exhibited by other group-living rodents such as naked mole rats [17].

Nowak et al. [6] challenged the monogamy hypothesis for social hymenoptera on two grounds. Specifically, the phylogenetic evidence is weak because the ancestral state of the majority of solitary hymenoptera species is likely to be monogamy. Hence, monogamy as an evolutionary preadaptation is indistinguishable from ecological factors favoring cooperation, such as defending nests or sequentially provisioning offspring. More broadly, the entire approach of inclusive fitness modeling may be rife with simplifying assumptions that produce spurious predictions. Rather than the actor-based approach of kin selection (e.g., do individuals have higher inclusive fitness for helping parents or raising their own offspring?), Nowak et al. [6] advocated a gene-based approach (e.g., can alleles for helping others reproduce invade non-cooperative populations?). They derived general cases showing that *r *values alone may not always accurately predict the evolution of cooperation. However, they gave no example of a specific behavioral or life history phenomenon where a kin selection model gives an erroneous prediction relative to a gene-based model [18].

The monogamy hypothesis is where kin selection possibly gets it wrong. If its basic premise of an inclusive fitness advantage to helping with high relatedness is correct, then the same result ought to occur when cooperation is modeled as the spread of a helping allele.

## Results

### Helping allele (A) is dominant

**Figure 1:**
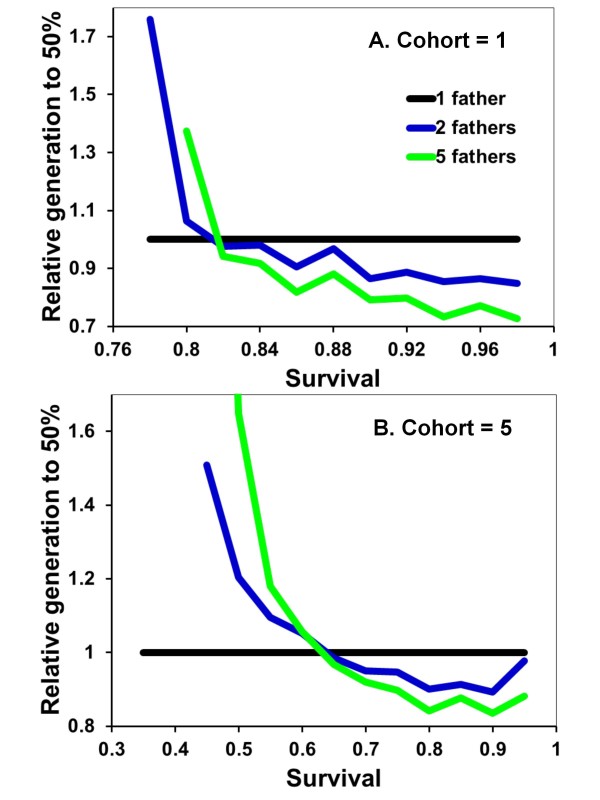
**Increase in helping as a dominant trait relative to mating strategy and expected survival of adults between cohorts**. The mean number of generations until the frequency of the helping allele equaled or exceeded 50% of the population are shown as ratios relative to single mating for 1 (black line), 2 (blue) or 5 (green) fathers (ratio = generations for *x *number of fathers/generations for 1 father). Where the blue and green lines exceed the black line, helping evolves faster with monogamy. Where they are less than the black line, helping increases more rapidly with polygamy. Regions of the graphs where individual lines do not extend indicate conditions where A never increased to the 50% criterion for the given numbers of fathers. Each point on the figure represents the mean of 100 individual simulations with those sets of values. Offspring are produced in cohorts of one (A) or five (B).

Cooperation evolves under intermediate values of adult survival between offspring cohorts. If *s *is less than 0.78 or 0.40 for cohort sizes of 1 or 5 respectively, helping never predominates in the population (Figure 1). Similarly, if *s *= 1 such that mothers do not die between cohorts, there is no selective advantage to making or being a helper. With no maternal mortality, helping behavior does not invade the population in the model (Figure 1).

Under conditions that do favor helping, the monogamy hypothesis is not confirmed by a gene-based approach: helping does not consistently evolve more quickly with monogamy (Figure 1). Under many conditions, the number of mates does not affect how rapidly helping spreads through the population. The gene-based modeling approach reveals the reason. Any aa female mating with multiple males increases her chances of gaining a helper. However, helpers aid a relatively smaller fraction of sibs with A. These two probabilities approximately cancel out. From the helping allele perspective, single mating is a high variance strategy that pays off big, but rarely. Multiple mating is a low variance strategy that works more often, but with reduced effect.

There are associated advantages and disadvantages with mating strategy that are separate from differences in sibling relatedness. An advantage for single mating is that helpers aid both alleles equally. For example, all daughters from aa x A matings are aA. Thus, the frequency of A in helpers equals the frequency of A in aided female siblings. In contrast, if aa females mate multiply with males of both genotypes, then the frequency of A is higher in helping females than in helped ones. Although A can increase in the population due to across-group selection, it decreases in frequency within its own group. When inter-cohort survival is low, this cost to multiple mating results in helping evolving faster with monogamy (Figure 1).

Multiple mating has an advantage that if mothers die, subsequent benefits nonrandomly accrue to A alleles. Only daughters having this allele can inherit nests. As inter-cohort survival increases, helping becomes more likely to evolve with polygamy and through groups of lower mean relatedness. Interestingly, polygamy is favored over a wider range of values than monogamy (Figure 1).

**Figure 2:**
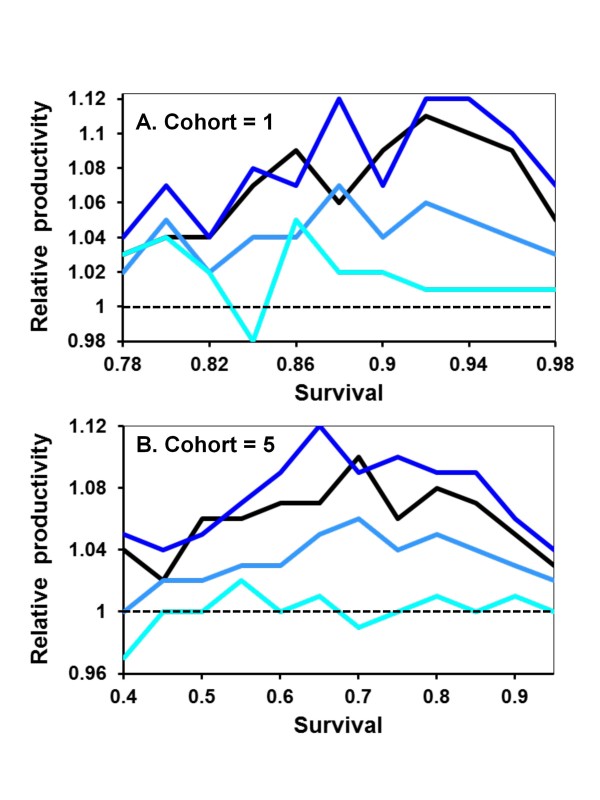
**Relative fitness of mating strategies as expected survival of adults between cohorts increases and with helping dominant**. The total numbers of offspring are summed across nests where females mate once or five times in populations at points when the frequency of the helping allele (A) first reaches 10 (darkest line), 25, 50, or 75% (lightest blue line) of the population. Values greater than one indicate that, on average, polygamous nests are more productive than monogamous nests. Each point represents the mean of 100 individual simulations. Offspring are produced in cohorts of one (A) or five (B).

Mating strategy is uniform across females within a population in the above results. However, when females are randomly assigned a number of mates (1 or 5) within the same population, multiply-mated nests consistently produce more offspring relative to singly-mated nests across a wide range of *s *values (Figure 2). The difference is particularly evident when A is the rarer allele. Only when A reaches high frequencies, such as 75% of the population, is nest productivity similar across mating strategies.

**Figure 3:**
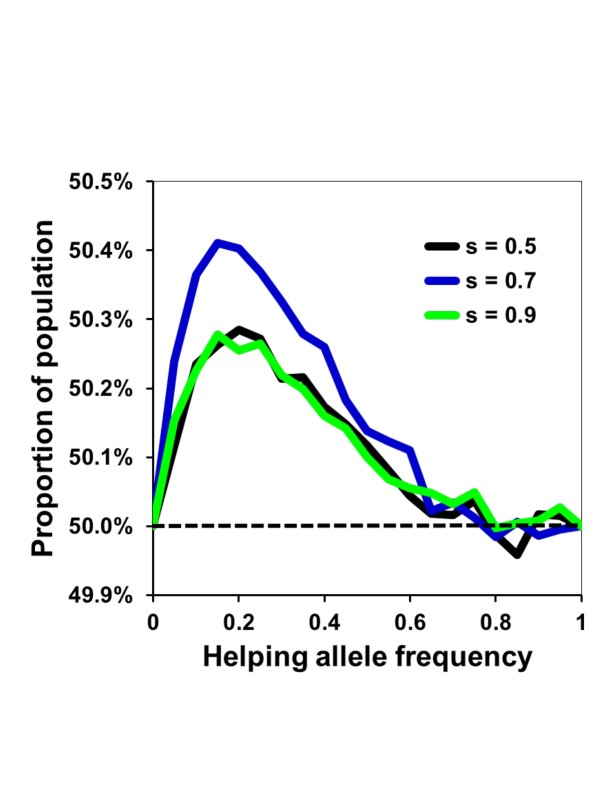
**Selection for multiple mating strategy**. An initial population of females was set with equal numbers of alleles for mating with one or five males at varying population frequencies of a dominant helping allele (A). Females reproduced offspring in cohorts of five with an expected survival (*s*) of 0.5, 0.7 or 0.9 between cohorts. The population percentages of alleles for multiple mating are shown after one generation of reproduction. Each point represents the mean of 10,000 individual simulation runs.

When mating strategies are themselves genetically determined, alleles for higher mating frequency are favored (Figure 3). Because polygamous nests produce more helping daughters, this in turn increases lifetime nest productivity (Figure 2) and the potential transmission of alleles for multiple mating. This advantage disappears at higher frequencies of the helping allele because all mating strategies are likely to produce helpers (Figure 3). Interestingly, with low survival values (e.g., *s *= 0.5) cooperation evolves much more rapidly in monogamous rather than polygamous populations (Figure 1B), but polygamous nests are on average more productive within populations (Figure 2B) and multiple mating alleles are at a selective advantage when helping is not ubiquitous (Figure 3). Therefore, the evolution of helping appears to always have the correlated effect of also favoring mating with multiple potential fathers.

### Helping allele (A) is recessive or males are diploid

**Figure 4:**
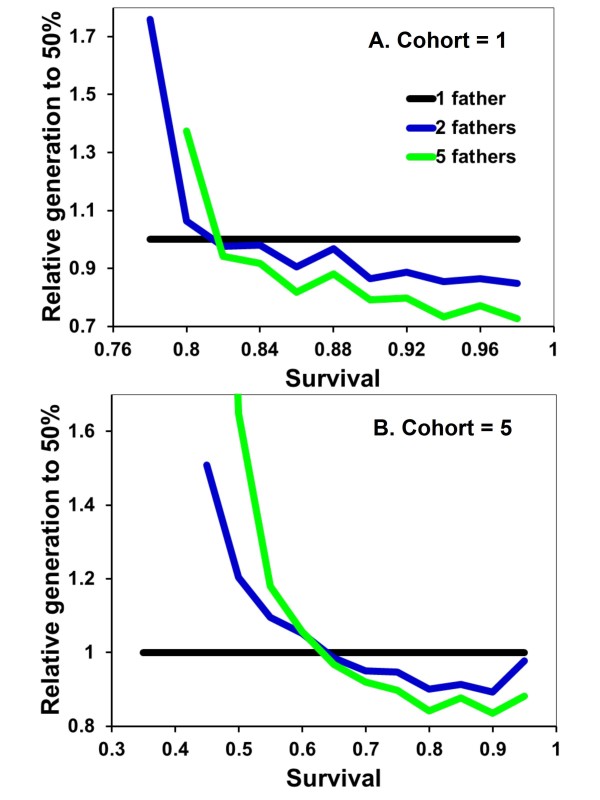
**Relative fitness of mating strategies as expected survival of adults between cohorts increases and with helping recessive**. Details are as given in Figure 1.

**Figure 5:**
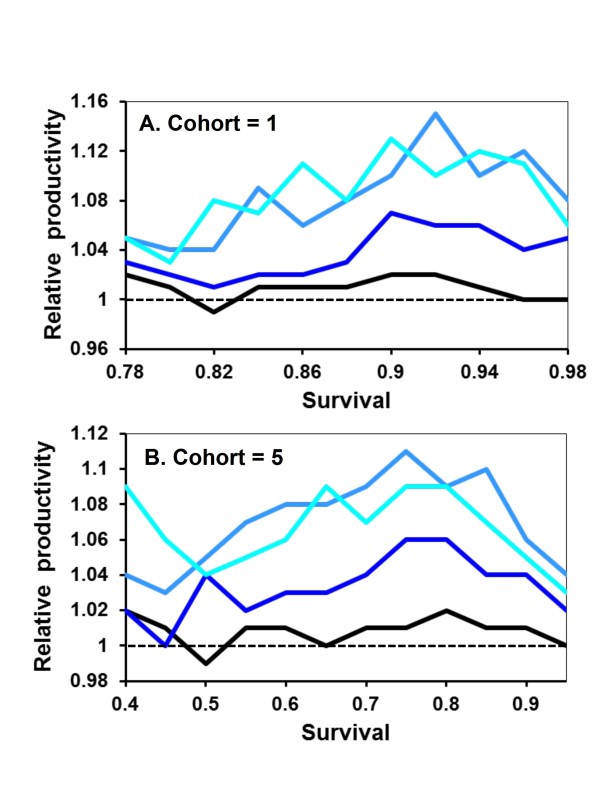
**Relative fitness of mating strategies when helping was recessive**. Details are as given in Figure 2.

**Figure 6:**
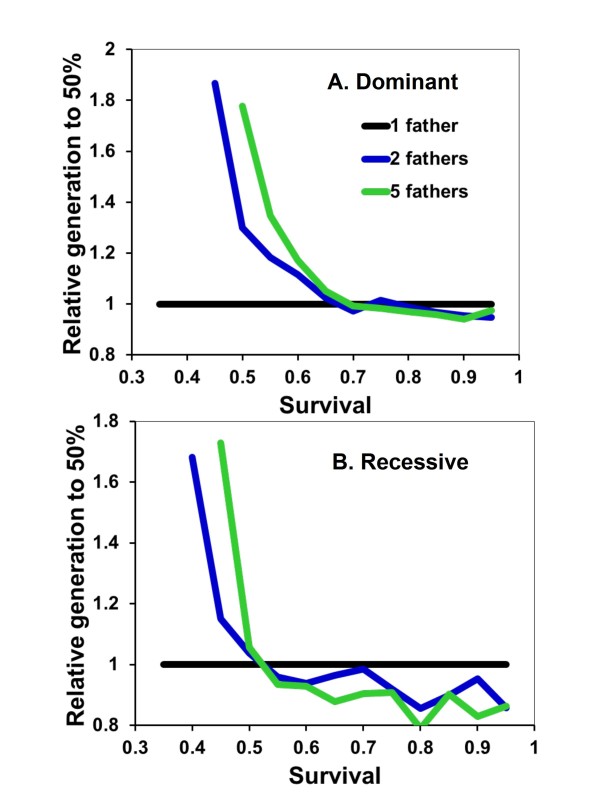
**Increase in helping relative to mating strategy and expected survival of adults between cohorts of five offspring in diploids**. Helping is either dominant (A) or recessive (B). The results are otherwise as described in Figure 1.

**Figure 7:**
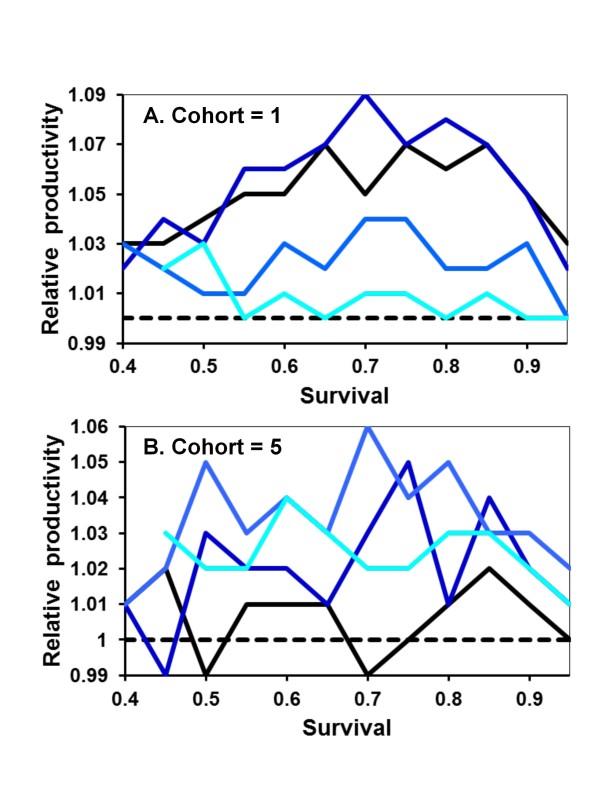
**Relative fitness of mating strategies as expected survival of adults between cohorts of five offspring increases in diploids**. Helping is either dominant (A) or recessive (B). The results are otherwise as described in Figure 2.

Helping behavior evolves more slowly when A is recessive because the allele must initially drift under neutral selection until its frequency is high enough that AA daughters start being produced. Apart from this, the results are broadly similar to when A is dominant (Figure 4). Monogamy is favored when *s *is low, equal to polygamy when *s *is intermediate, and disfavored when *s *is high. Also similar to when A is dominant, monogamous nests are, on average, less productive when A is rare in the population (Figure 5). Recessive alleles differ in that multiple mating has the greatest relative selective advantage at intermediate frequencies of A. This is again likely due to helping rarely expressed at low frequencies of recessive A alleles. Assuming diploid males has no qualitatively different effects on the results and conclusions (Figure 6 &7).

### Indirect fitness and multiple helpers

**Figure 8:**
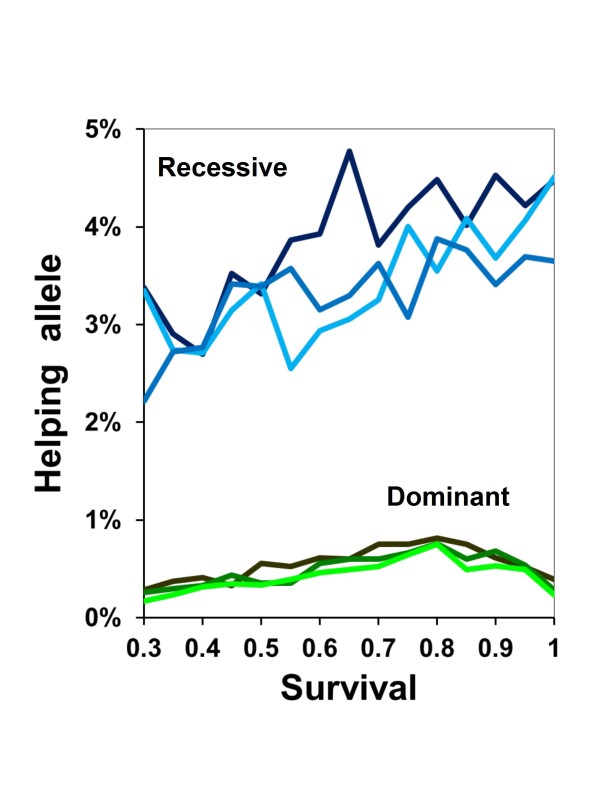
**Effect of indirect fitness and kinship on helping**. When helpers are randomly redistributed across nests, the frequencies of A after 500 generations are not above levels due to mutation rate for any level of expected survival of adults between cohorts. Helping is either dominant (green lines: darkest = 1 father, lightest = 5 fathers) or recessive (blue). Each point represents the mean of 100 individual simulations.

When helpers randomly drift across nests, kin and non-kin are equally likely to benefit from survival insurance. In the absence of indirect fitness gains for biasing survival insurance towards kin, A does not increase in frequency beyond levels produced by mutation and genetic drift (Figure 8). Across every simulation with all levels of adult survival, the A allele never reached a frequency of 50% or more in the population.

Allowing multiple helpers, each of which provides an identical increase in nest survivorship, does not result in helping spreading more rapidly with monogamy than polygamy. Under conditions where A is dominant and offspring come in cohorts of five, the helping allele reaches a population frequency of 50%, on average, in 56.7, 35.0 or 23.8 generations (with *s *= 0.7, 0.75, or 0.8, respectively) with monogamy. In comparison, the helping allele reaches the 50% criterion in 37.2, 23.9 and 18.7 generations when females mate with two males. When mating with five males, the 50% criterion is attained in 22.4, 18.4 and 15.6 generations. Thus across this range of comparison, the 50% criterion is reached in 39-79% of the number of generations required under monogamy. Contrasted to the results with only one helper (Figure 1B), polygamy is even more conducive to the spread of helping behavior when multiple helpers increase nest survival in an additive, linear fashion.

## Discussion

Monogamous behavior on the part of females in mating with only one male does not appear to generally act as a spring-loaded preadaptation for the evolution of cooperative breeding. To the contrary, polygamy through polyandry is more likely to be particularly beneficial. Although phylogenetic signals may correlate monogamy with eusociality and reproductive skew [13-16], such relationships are not *prima facie *support for kin selection theory. Other shared life history traits apart from mating behavior must be reconsidered as predisposing factors for evolution of cooperative breeding [6]. Indeed, the more interesting message in the phylogenetic data may be how often social systems of low relatedness have evolved (e.g., becoming polygynous or polyandrous) from high-relatedness ancestral species. For example, polyandry appears to have evolved independently from monoandry in 22 clades of ants [15]. Groups of low relatedness are likely to have correlated benefits arising from increased group-level genetic diversity, and it appears these benefits often exceed the evolutionary advantages of high within-group relatedness [9].

The contradiction of the monogamy hypothesis by a gene-based model supports criticism that inclusive fitness models are potentially inaccurate [6]. There are two reasons, however, why these results do not contradict kin selection, *per se*. First, comparing rearing offspring versus rearing full or half sibs in deriving the monogamy hypothesis is overly simplistic. This fails to include a long chain of probabilities of outcomes. For example, the true fitness payoff for a daughter in helping must include: the probability of surviving her mother across each potential remaining offspring cohort; if she does survive her mother, the probability that she herself will recruit a helper (which will depend on her mating strategy and the current frequency of A in the population); the subsequent probability(ies) that she will in turn be superseded by her daughter; etc. This value must be compared to a similarly extensive calculation of fitness for leaving. Note also that this is from daughter's 'point of view'. The payoffs would be different if asking whether the mother should manipulate a daughter into helping. Thus a gene-based model might indeed predict the same result as a fully comprehensive inclusive fitness model. Nevertheless, a gene-based approach is clearly a much simpler and more robust approach to this question. The stochastic simulations include every possible series of outcomes weighted by their probability of occurrence. None of these have to be calculated *a priori*. The model also has no 'point of view' that requires specifying the differential effects on parent and offspring fitness. If costs to offspring exceed benefits for parents (or vice versa), then reproductive suppression will not be selectively advantageous.

The second reason is more fundamental than a choice of analytical approaches. Although helping often increases more rapidly with lowered relatedness in the gene-based model, positive kin assortment is still required. Even with five fathers, relatedness would not be random (i.e., *r *> 0, with *r *being a measure of positive assortment such that members of the same nest are more likely to share the A allele than individuals drawn randomly from the population). Therefore, helpers can gain both direct fitness from inheriting their mother's position as reproducing adult and indirect fitness through assuring the survival of half and full sibs. Randomly redistributing helpers as if they were drifting to join nests creates a situation where the helpers have a mean expectation of *r *= 0 in terms of kin assortment to the present brood. Helping, therefore, can be selected only through direct fitness gains from future nest inheritance. Removing any potential for kin selection prevents helping from increasing in frequency (Figure 8). Thus, a gene-based approach is also a variation on Hamilton's rule. The basic principle remains unchanged: reproductive altruism evolves only when kin are sufficiently and differentially benefitted.

## Conclusion

Gene-based models may be superior to actor-based, kin selection models with variance in outcomes or when fitness consequences vary across interacting individuals. Thus more clearly than an inclusive fitness approach, the gene-based approach demonstrates that monogamy does not intrinsically create conditions predisposed for the evolution of helping. Gene-based approaches have also successfully modeled the evolution of genetic diversity through across-individual epistatic effects (i.e., social heterosis [9,19]). One must not, however, confuse the utility of a particular mathematical approach with the validity of the underlying evolutionary theory. Showing low relatedness is selectively advantageous to high relatedness is not equivalent to concluding that relatedness is unimportant. Actor-based models of kin selection have had a long and fruitful history of framing interesting evolutionary questions [2-5] and will undoubtedly continue to be successful in explaining many features of life history. Kin selection theory is important because kinship mattered throughout evolutionary history. The key point is that genetic relatedness and genetic diversity are inversely correlated and the evolutionary consequences of this correlation are what future modeling endeavors should aim to better understand.

## Methods

The model is written in TrueBasic^®^, with code provided in Additional File 1 for the main model. Populations were initially modeled as haplodiploid with a wasp-like life history. In the first generation 200 solitarily-nesting females, all homozygous for non-helping (genotype aa), mated to either 1, 2, or 5 males. To be consistent with terminology used elsewhere, departures from monogamy are equated only with mating status (monoandry versus polyandry). I do not consider the case of multiple females breeding simultaneously (i.e., polygyny in social insects). Each mother could produce a maximum of 50 offspring. Offspring were produced sequentially either one at a time or in groups of five. Sex of each offspring was randomly chosen and equally likely to be female or male. Between each successive reproductive event, mothers had an expected survival rate of *s *(varied between 0.3 to 1.0, depending on the simulation). Offspring survival required at least one adult on the nest until their cohort matured. Without an adult, any remaining offspring died. Mature offspring either became helpers or were seeded into a common reproductive pool for the next generation.

For each offspring, the maternal allele contribution was randomly chosen with equal probability. If the offspring was female, the father was randomly chosen from the mother's mates. All sons, irrespective of genotype, became reproductives. All aa daughters (and aA daughters if A was recessive) became reproductives. The first aA daughter produced (AA if recessive) became a helper on the nest. The only exception was for the last possible cohort (i.e., where the nest reaches the maximum of 50 offspring). At this point, there is no possibility to provide help and all daughters were added to the common reproductive pool. Although this behavior is framed as beneficial 'helping' from a daughter, the model is the same if A is defined as a susceptibility to being manipulated into remaining by the mother. Neither mothers, fathers nor helpers were added to the common reproductive pool. Each generation was a new sample of potential parents and helpers.

Helping arose in the form of mutation from allele a to A. The mutation rate was 0.001 per generation for every allele in sons and non-helper daughters. Because the main variable of interest was the rate at which helping spread due to mating strategy, back mutations were not relevant and, therefore, were not considered in the simulations.

Only one helper could be present on a given nest at a time in most simulations because survival benefits tend to rapidly decline with helper number [20]. The helper's inter-cohort survival rate was the same as her mother's. This assumes there are no 'queen-like' preadaptations in mothers to reduce foraging or otherwise increase their own survival rates. Helpers did not reproduce in the presence of their mother, and mothers did not increase cohort size or maximum cohort numbers with helpers. These assumptions follow the headstart or assured fitness return models of cooperation where the helper's benefit for the mother and her siblings is for potential nest survival if the mother should die [21,22]. If both mother and helper were present, all other daughters entered the common reproductive pool. If the mother survived and the helper died during some interval, a new helper could be recruited. If the mother died and the helper survived, the helper became the new mother on the nest and mated with the same number of males as her mother. The following cohort of offspring was still the previous mother's, but the subsequent cohort was the offspring of the promoted helper. Again, a new helper could be potentially recruited. Sequential monogyny with the death and replacement of a single dominant egg-layer are common life histories in primitively eusocial species [23]. Nest inheritance and mating by helpers created totipotence for all individuals in the model. There was no permanently sterile caste, and as such this appears to violate the initial premise of the monogamy hypothesis. Boomsma [5,6] proposes that a 'monogamy window' is evolutionarily needed for transition from cooperative breeding to full eusociality with division of labor across castes in social insects. However, even in highly eusocial species, workers will often reproduce after the death of the queen [24,25], which was what happened in this model. Thus the reproductive life history modeled here would apply to both cooperatively-breeding and eusocial species.

The next generation was initiated after reproduction was tallied across all nests. Two hundred new females and the appropriate number of mates were randomly chosen from the common reproductive pool. This pool was then discarded, to be filled again from the new generation of nests. Each simulation ran until either a predetermined frequency of helping was reached (e.g., A composes 50% of the population). If the target frequency level was not reached in 500 generations, the simulation was terminated with the conclusion that helping was selectively disadvantaged. Each set of conditions was simulated 100 times. One complication was that as A reaches higher frequencies, drawing out females to be daughter helpers biases the reproductive pool towards males. To counter this, I included an increasing initial sex biasing factor such that each offspring produced had a slightly increased probability of being female (e.g., 52-54% female). Because most of the analyzed results examine the initial change in the frequency of A, the biasing correction factor has a trivial effect. The simulations were repeated as described under cases where A is recessive or males are diploid. In the latter case, each offspring had a randomly chosen father (if mothers were polygamous), and its contributed allele to the offspring was randomly chosen.

The above simulations compared the rate of increase of helping in populations that differed in the number of matings per female. Within populations, all females mated the same number of times. To examine within-population advantages for mating number, I used two methods to compare the fitness. In both maternal mating strategy was randomly assigned as having 1 or 5 mates. Daughters that inherited nests mated with the same number of males as their mother. In the first method, fitness was calculated as the total number of offspring produced on a nest where females mated either once or five times. This method determined whether monogamous or polygamous nests were intrinsically more productive as helping spread through the population. However, differences in reproductive output may not necessarily translate to differential selection on mating number itself because mating preference was not heritable. Therefore in a second method, I started with populations having fixed frequencies of a dominant A allele ranging between 0.05 and 0.95, randomly distributed across individuals. In this case mothers also varied genotypically for mating strategy with alleles specified for mating with 1 or 5 males. At the beginning of each simulation, each allele was set at 50% of the initial population. A mother's expressed mating strategy was the rounded average of her two alleles (e.g., a mother with alleles for mating once or five times, mated with three males). Nests reproduced as described above for one generation and alleles contributed to the next generation's reproductive pool were tallied for each mating strategy.

Another model variation eliminated the possibility of helpers gaining indirect fitness by helping kin. In these simulations, all individuals that became helpers were randomly distributed across surviving nests for every cohort of offspring produced across all mothers. Biologically, this is as if helpers drift within the population and randomly associate with any other nest. In this situation the average helper is unrelated to the individuals on the nest she joins. Although such a system of completely random, drifting helpers has never been observed, this variation serves an important function for understanding the model results. In the above scenarios, helping has both indirect fitness returns (i.e., assuring survival of non-descendant kin) and direct fitness (i.e., the possibility of inheriting a nest and rearing own offspring). With drifting helpers, only direct fitness returns to the potential helper can select for helping behavior.

A final variation of the model allowed up to four helpers to be simultaneously present on a nest. To make the benefit of helping equal from the first to the fourth helper, nest survival was increased by a constant 5% for every helper present. Therefore, if a nest with only the mother present had a 70% intercohort survival probability (*s*), the same nest with four helpers would have a 90% survival probability. The design is such that fitness benefits of multiple helpers accrues linearly. The mother's intercohort survival rate was equal to her survival rate by herself. Thus, daughters inherited the nest with the same probability as in the previous simulations. Nest inheritance was always by the oldest helper present. In this variation, A was only considered as a dominant allele, offspring came in cohorts of five, and *s *ranged from 0.7-0.8.

## Supplementary Material

Additional file 1 **BMC Model helping% Figure **1 &4. The code, written in TrueBasic, for the evolution of helping behavior under the initial assumptions that generate Figure 1 and 4. Click here for file
