# Supplementary material for: Monogamy and high relatedness do not preferentially favor the evolution of cooperation
Source: BMC Evol Biol. 2011 Mar 4;11:58. doi: 10.1186/1471-2148-11-58 (PMC3058046; doi:10.1186/1471-2148-11-58)
Supplement: Additional file 1 — BMC Model helping% Figure 1 &4. The code, written in TrueBasic, for the evolution of helping behavior under the initial assumptions that generate Figure 1 and 4. [file 1471-2148-11-58-S1.DOCX]

!PROGRAM TO DETERMINE # OF GENERATIONS FOR A HELPING ALLELE TO REACH 50% OF THE POPULATION

!GENERATES DATA USED TO MAKE FIGURES 1 & 4 IN NONACS MANUSCRIPT

!MODEL IS WRITTEN IN TRUEBASIC

LET mothers = 200 !# OF NESTS EACH WITH ONE MOTHER

LET mutate = 0.001 !MUTATION RATE OF a TO A (W/O BACK MUTATIONS)

LET gmax = 500 !MAXIMUM # OF GENERATIONS

LET smax = 100 !# OF SIMULATIONS

LET offspring = 50 !MAXIMUM # OF OFFSPRING PER NEST

LET printon = 0 !CREATE OUTPUT FILE (1 = YES)

LET queue = 1 !1=HELPER REPRODUCES AFTER MOTHER DEATH; 2 = DOES NOT, ALL FUTURE OFFSPRING ARE MOTHERS

LET cmin = 0.1 !INITIAL CRITICAL FREQUENCY OF HELPING ALLELES AT WHICH SIMULATION STARTS RECORDING DATA

LET cmax = 0.5 !MAXIMUM HELPING FREQUENCY AT WHICH LOOPS STOP

RANDOMIZE !SEEDS NEW RANDOM NUMBERS FOR EACH MODEL RUN

!CREATE AND REDIMENSION NEEDED ARRAYS

DIM mom(1,1) !ARRAY FOR DATA ON REPRODUCTIVE FEMALES ON NEST

DIM Fpool(1,1) !FEMALE OFFSPRING POOL

DIM Mpool(1) !MALE OFFSPRING POOL

DIM Mpool1(1) !MALE OFFSPRING POOL FOR CHOOSING HELPER MATES

DIM survive(2) !IS MOM AND HELPER ALIVE?

DIM TgenFreq(1,1) !RECORDS GENERATION AT WHICH FREQUENCY OF A PASSES CRITICAL FREQUENCY

!SET MAXIMUM POTENTIAL # OF OFFSPRING AND REDIMENSION ARRAYS

LET females = offspring * mothers

LET males = offspring * mothers

MAT REDIM Fpool(females,2)

MAT REDIM Mpool(males)

MAT REDIM Mpool1(males)

MAT REDIM TgenFreq(3,smax)

FOR cohort = 1 to 5 step 4 !SIZE OF OFFSPRING COHORT

IF cohort = 1 then !FILENAME RECORDS SIMULATION VALUES

LET SSS$ = "74to100qc1"

LET s1 = 0.74 !MIN TO MAX INDIVIDUAL SURVIVAL

LET s2 = 1.001

LET sstep = 0.02

ELSE

LET SSS$ = "30to100qc5"

LET s1 = 0.30 !MIN TO MAX INDIVIDUAL SURVIVAL

LET s2 = 1.001

LET sstep = 0.05

END IF

!CREATE FILES FOR OUTPUTS THAT CAN BE OPENED IN EXCEL

IF printon = 1 then

OPEN #2: name "Helper@NestC_" & SSS$, create newold, access outin, organization text

SET #2: MARGIN 500

PRINT #2: "Survive"; ","; "D or R"; ","; "Fathers"; ","; "Sim #"; ","; "C%"; ","; "Generation"; ","; "SD"

END IF

FOR s = s1 to s2 step sstep !RANGE OF ADULT SURVIVAL BETWEEN OFFSPRING COHORTS

FOR express = 1 to 2 !1 = TRAIT IS DOMINANT; 2 = RECESSIVE

FOR fathermax = 1 to 3 !NUMBER OF MATES FOR FEMALE

IF fathermax = 3 then LET fathermax = 5

PRINT "Cohort", "Survive", "D or R", "Fathers", "Sim #", "C%", "Generation", "SD"

!REDIMENSION AND EMPTY NEEDED ARRAYS

MAT REDIM mom(mothers,3+fathermax)

MAT TgenFreq = 0

FOR sim = 1 to smax !# OF SIMULATIONS PER SET OF MODEL VALUES

!SET MAXIMUM POTENTIAL # OF OFFSPRING

LET females = offspring * mothers

LET males = offspring * mothers

!CREATE INITIAL POOL OF GENOTYPES OF FEMALES AND MALES

FOR i = 1 to females

IF rnd < mutate then LET Fpool(i,1) = 1 else LET Fpool(i,1) = 0 !1 = HELPING ALLELE; 0 = NULL, NON-HELPING ALLELE

IF rnd < mutate then LET Fpool(i,2) = 1 else LET Fpool(i,2) = 0

IF rnd < mutate then LET Mpool(i) = 1 else LET Mpool(i) = 0

NEXT i

LET cper = 0 !INITIAL FREQUENCY OF HELPING ALLELE

LET picksex = 0.5 !INITIAL LIKELIHOOD OF FEMALE OR MALE OFFSPRING

LET ccrit = cmin !CRITICAL FREQUENCY OF HELPING ALLELES AT WHICH SIMULATION STARTS RECORDING DATA

FOR gen = 1 to gmax

LET Wallele = 0 !COUNTER FOR NON-HELPING NULL ALLELES

LET Callele = 0 !COUNTER FOR HELPING ALLELES

!RESET NEEDED ARRAYS

MAT mom = (-99)

MAT Mpool1 = Mpool

!PICK MOTHERS RANDOMLY FOR NEXT GENERATION

LET fcount = 0

FOR i = 1 to mothers

LET fpick = int(females*rnd) + 1

!RECORD MOTHERS DIPLOID GENOTYPE

LET fcount = fcount + 1

LET mom(fcount,1) = Fpool(fpick,1)

LET mom(fcount,2) = Fpool(fpick,2)

!PICK FATHERS RANDOMLY TO MATE WITH MOTHER i

LET mcount = 0

FOR j = 1 to fathermax

LET mpick = int(males*rnd) + 1

!RECORD FATHERS HAPLOID GENOTYPE

LET mcount = mcount + 1

LET mom(fcount,2+mcount) = Mpool(mpick)

NEXT j

LET mom(fcount,3+fathermax) = fathermax !RECORD # OF MATES FOR MOTHER i

NEXT i

!EMPTY OFFSPRING POOLS FOR NEW GENERATION

MAT Fpool = (-99)

MAT Mpool = (-99)

!SET REQUIRED COUNTERS AND DATA COLLECTORS TO ZERO

LET fcount = 0

LET mcount = 0

!REPRODUCTION FOR EACH NEST

FOR i = 1 to mothers

!SET MOTHER IS PRESENT, HELPER IS NOT FOR 1ST COHORT

LET survive(1) = 1

LET survive(2) = 0

LET helper = 0

!REPRODUCE OFFSPRING BY COHORTS

FOR j = 1 to offspring/cohort

!DETERMINE ADULT SURIVAL (ASSUMES MOTHER ALWAYS SURVIVES TO PRODUCE 1ST COHORT)

IF j > 1 then

IF rnd > s then LET survive(1) = 0 !DID MOTHER DIE?

IF rnd > s then LET survive(2) = 0 !DID HELPER DIE?

IF survive(2) = 0 then LET helper = 0

END IF

!IF AN ADULT IS PRESENT, REPRODUCE A COHORT

IF survive(1) = 1 or survive(2) = 1 then

!DETERMINE SEX AND ROLE OF EACH OFFSPRING

FOR jj = 1 to cohort

IF rnd >= picksex then !OFFSPRING IS A FEMALE

!RANDOMLY PICK ALLELE CONTRIBUTED FROM MOTHER & FATHER

IF rnd < 0.5 then LET daughter1 = mom(i,1) else LET daughter1 = mom(i,2)

LET fpick = int(rnd*fathermax) + 3

LET daughter2 = mom(i,fpick)

!MAKE DAUGHTER A HELPER, IF THIS IS NOT LAST COHORT, NO HELPER IS PRESENT & DAUGHTER HAS CORRECT GENOTYPE

IF helper = 0 and j < offspring/cohort then

IF express = 1 then !TRAIT IS DOMINANT

IF daughter1 = 1 or daughter2 = 1 then !DAUGHTER BECOMES HELPER

LET survive(2) = 1 !HELPER IS NOW PRESENT

!RECORD NEST AS HAVING HELPER AND HELPER'S GENOTYPE

LET helper = 1

LET dmom1 = daughter1

LET dmom2 = daughter2

ELSE !DAUGHTER DOES NOT BECOME HELPER, ADD TO NEXT GENERATION'S POTENTIAL MOTHERS

LET fcount = fcount + 1

LET Fpool(fcount,1) = daughter1

LET Fpool(fcount,2) = daughter2

!POTENTIALLY MUTATE ALLELE FROM NULL TO HELPING (NO BACK MUTATIONS)

IF rnd <= mutate then LET Fpool(fcount,1) = 1

IF rnd <= mutate then LET Fpool(fcount,2) = 1

!COUNT ALLELE TYPES

IF Fpool(fcount,1) = 0 then LET Wallele = Wallele + 1 else LET Callele = Callele + 1

IF Fpool(fcount,2) = 0 then LET Wallele = Wallele + 1 else LET Callele = Callele + 1

END IF

ELSE !TRAIT IS RECESSIVE

IF daughter1 = 1 and daughter2 = 1 then

LET survive(2) = 1

LET helper = 1

LET dmom1 = daughter1

LET dmom2 = daughter2

ELSE

LET fcount = fcount + 1

LET Fpool(fcount,1) = daughter1

LET Fpool(fcount,2) = daughter2

IF rnd <= mutate THEN LET Fpool(fcount,1) = 1

IF rnd <= mutate THEN LET Fpool(fcount,2) = 1

IF Fpool(fcount,1) = 0 then LET Wallele = Wallele + 1 else LET Callele = Callele + 1

IF Fpool(fcount,2) = 0 then LET Wallele = Wallele + 1 else LET Callele = Callele + 1

END IF

END IF

ELSE !DAUGHTER DOES NOT BECOME HELPER, ADD TO NEXT GENERATION'S POTENTIAL MOTHERS

LET fcount = fcount + 1

LET Fpool(fcount,1) = daughter1

LET Fpool(fcount,2) = daughter2

IF rnd <= mutate then LET Fpool(fcount,1) = 1

IF rnd <= mutate then LET Fpool(fcount,2) = 1

IF Fpool(fcount,1) = 0 then LET Wallele = Wallele + 1 else LET Callele = Callele + 1

IF Fpool(fcount,2) = 0 then LET Wallele = Wallele + 1 else LET Callele = Callele + 1

END IF

ELSE !OFFSPRING IS A MALE

!RANDOMLY DETERMINE SON'S HAPLOID GENOTYPE

IF rnd < 0.5 then LET son = mom(i,1) else LET son = mom(i,2)

!ADD SON TO NEXT GENERATION'S POTENTIAL FATHERS

LET mcount = mcount + 1

LET Mpool(mcount) = son

!POTENTIALLY MUTATE ALLELE FROM NULL TO HELPING (NO BACK MUTATIONS)

IF rnd <= mutate then LET Mpool(mcount) = 1

IF Mpool(mcount) = 0 then LET Wallele = Wallele + 1 else LET Callele = Callele + 1

END IF

NEXT jj

!IF MOTHER DIES

IF survive(1) = 0 and queue > 0 then

!FORMER HELPER MATES AND WILL REPRODUCE NEXT COHORT; OTHERWISE ALL COHORTS FROM ORIGINAL MOTHER

IF queue = 1 then

!REPLACE MOM'S GENOTYPE WITH HELPER'S

LET mom(i,1) = dmom1

LET mom(i,2) = dmom2

!MATES HELPER W. SAME # NUMBER OF FATHERS AS MOTHER

LET mcount1 = 0

!CHOOSE MATES FROM PREVIOUS POOL OF AVAILABLE MALES

FOR k = 1 to fathermax

LET mpick = int(males*rnd) + 1

LET mcount1 = mcount1 + 1

LET mom(i,2+mcount1) = Mpool1(mpick)

NEXT k

END IF

!ALLOWS NEW HELPERS

LET survive(1) = 1

LET survive(2) = 0

LET helper = 0

END IF

ELSE !NO ADULT ALIVE, END REPRODUCTION ON THIS NEST

LET j = offspring/cohort

END IF

NEXT j

NEXT i

!RECORDS NUMBER OF FEMALES AND MALES IN NEXT GENERATION'S POTENTIAL REPRODUCTIVES

LET females = fcount

LET males = mcount

!REBALANCES SEX RATIOS, TO SLIGHTLY FAVOR RARER SEX: CORRECTS FOR DRAWING OUT FEMALES AS HELPERS

LET sex = fcount / (fcount + mcount)

IF sex < 0.5 then LET picksex = picksex - 0.01 ELSE LET picksex = picksex + 0.01

!NEW FREQUENCY OF HELPING ALLELE

LET cper = Callele / (Callele + Wallele)

!RECORD DATA IF THRESHOLD IS REACHED

IF cper >= ccrit then

!SET NEW HIGHER VALUE FOR NEXT DATA RECORD

IF ccrit = 0.5 then

LET TgenFreq(3,sim) = gen

LET ccrit = 0.75

END IF

IF ccrit = 0.25 then

LET TgenFreq(2,sim) = gen

LET ccrit = 0.5

END IF

IF ccrit = 0.1 then

LET TgenFreq(1,sim) = gen

LET ccrit = 0.25

END IF

END IF

!END RUN IF HELPING FREQUENCY CROSSES MAXIMUM THRESHOLD

IF cper >= cmax then LET gen = gmax

NEXT gen

!FILL IN DATA VALUES IF THRESHOLDS WERE NOT REACHED DURING RUN

IF ccrit = 0.1 then

LET TgenFreq(1,sim) = gmax

LET ccrit = 0.25

END IF

IF ccrit = 0.25 then

LET TgenFreq(2,sim) = gmax

LET ccrit = 0.5

END IF

IF ccrit = 0.5 then LET TgenFreq(3,sim) = gmax

NEXT sim

!CALCULATE MEAN & SD FOR GENERATIONS TO 50% OF ALLELE A

FOR i = 1 to 3

LET y = 0

LET y2 = 0

FOR j = 1 to smax

LET y2 = y2 + TgenFreq(i,j)^2

LET y = y + TgenFreq(i,j)

NEXT j

LET y = y / smax

LET sd = ((y2 - smax*y^2) / (smax - 1)) ^ 0.5

IF i = 1 then LET ccc = 0.1

IF i = 2 then LET ccc = 0.25

IF i = 3 then LET ccc = 0.5

!PRINTING COMMANDS

IF printon = 1 then PRINT #2: s; ","; express; ","; fathermax; ","; smax; ","; ccc; ","; round(y,2); ","; round(sd,2)

PRINT cohort, s, express, fathermax, smax, ccc, y, sd

NEXT i

NEXT fathermax

NEXT express

NEXT s

!CLOSE PRINTING

IF printon = 1 then CLOSE #2

NEXT cohort

END
